# Epidemiology and survival of cervical cancer in the French West-Indies: data from the Martinique Cancer Registry (2002–2011)

**DOI:** 10.1080/16549716.2017.1337341

**Published:** 2017-06-26

**Authors:** K. Melan, E. Janky, J. Macni, S. Ulric-Gervaise, M.-J. Dorival, J. Veronique-Baudin, C. Joachim

**Affiliations:** ^a^ Martinique Cancer Registry, AMREC, Fort-de-France, Martinique; ^b^ Gynecology Obstetrics Department, University Hospital of Pointe-à-Pitre, Pointe-à-Pitre, Guadeloupe; ^c^ Oncology Haematology Urology Pathology Department, University Hospital of Martinique, Fort-de-France, Martinique; ^d^ Laboratoire de Pathologie SERAL, Fort-de-France, Martinique

**Keywords:** Cervical cancer, survival, epidemiology, spatial distribution, population-based, cancer registry

## Abstract

**Background**: The Caribbean ranks seventh among world regions most affected by cervical cancer. Social health inequalities, such as differences in access to screening services, engender disparities in incidence and mortality between low- and middle-income countries and industrialized countries. The French National Cancer Plan 2014–2019 focuses on reducing inequalities in cervical cancer.

**Objective**: The aim of this study was to describe the geographical distribution and overall survival of cervical cancer, based on data from a population-based cancer registry in Martinique (French West-Indies).

**Methods**: We included all cases of cervical cancer diagnosed between 2002 and 2011. The geographical distribution was described by zone of residence and by aggregated units for statistical information (IRIS). Based on the results of the model, standardized incidence rates (SIRs) were calculated using a Gamma Poisson model. Survival rates were calculated using the Kaplan–Meier method. Cox proportional hazards models were used to investigate the risk factors for cervical cancer mortality.

**Results**: A total of 1253 cases were analyzed (947 in situ tumors and 306 invasive cancers). 1230 cases with geolocalization were used to map the distribution of the incidence of in situ and invasive cervical cancers. Five IRIS were significantly over-incident. The 5-year overall survival rate was 55%, with a median survival of 6.5 years [95% CI: 4.9–10.1]. Multivariate analysis confirmed age at diagnosis (HR = 2.15 [1.50–3.09]; p < 0.0001), FIGO stage (HR = 3.53 [2.50–4.99]; p < 0.0001) and zone of residence (HR = 1.51 [1.06–2.13]; p = 0.02) as risk factors.

**Conclusions**: Prognostic factors suggest that cervical cancer needs to be diagnosed at an early stage. Our results could allow cervical cancer screening programs to clearly identify geographical areas that would benefit from targeted interventions with a view to reducing incidence and mortality of cervical cancer in the Caribbean.

## Background

Around 266,000 women die worldwide from cervical cancer every year. It is the fourth most common female cancer in terms of incidence, with an estimated 528,000 new cases in 2012. Cervical cancer remains the most common cancer in women in Eastern and Middle Africa [1]. The majority of these deaths could have been prevented by existing prevention programs (screening, vaccination). In developed countries, cervical cancer incidence and mortality have declined by half over the last 30 years [1]. Improvements in hygiene and living conditions, as well as the introduction 50 years ago of a cytological screening test (the Pap smear test), have helped to decrease incidence and mortality of this type of cancer. Social health inequalities, such as differences in access to screening services and quality of treatment, engender disparities in incidence and mortality between low- and middle-income countries and industrialized countries. Because of its slow progression and the existence of many precancerous treatable lesions, this cancer is an ideal candidate for screening programmes, and could potentially become a rare disease in France [2]. Nonetheless, 3000 new cases of cervical cancer are detected in France each year, and 1100 women die from it [3].

The French National Cancer Plan 2014–2019 focuses on the development of organized screening throughout the country, with a view to reducing inequalities in cervical cancer [4]. Cervical cancer mortality rates are three times higher in Latin America and in the Caribbean than in North America, highlighting inequities in health [5]. The Caribbean ranks seventh among world regions most affected by cervical cancer [6]. The incidence of cervical cancer in the French West-Indies is higher than in metropolitan France [7]. In Martinique, cervical cancer is the fourth most prevalent cancer in terms of incidence in women, with 5.8% of solid tumors [8]. Because of this high level of incidence, Martinique implemented a pilot screening program in 1991 [9]. Several characteristics that are specific to the Caribbean (e.g. high incidence, type-specific human papilloma virus (HPV) distribution and socio-economic disparities) could lead to epidemiological and survival differences for this type of cancer. The aim of this study was therefore to describe the geographical distribution and overall survival of cervical cancer, based on data from a population-based cancer registry in Martinique (French West-Indies).

## Methods

### Patient and data sources

This retrospective study included all cases of cervical cancer diagnosed between 1 January 2002 and 31 December 2011. Cases of lymphoma were excluded. Patients were identified through the Martinique Cancer Registry database. Since 1981, this registry has collected data on cancer incidence and has participated in the national program for epidemiological surveillance of cancer in France, under the auspices of the French Network of General and Specialized Registries (FRANCIM). This study used population census data from the French National Institute for Statistics and Economic Research for the calculation of the incidence results.

Socio-demographic data and information on tumor characteristics (histological type, topography, stage at diagnosis [localized: stage I–II, advanced: stage III–IV], FIGO [International Federation of Gynecology and Obstetrics] stage, tumor size and invasion) were collected. Data on professional activity (active vs inactive) were also recorded. Active professional activity covered those actively engaged in the workforce either through outside or self-employment; inactive professional activity covered retirees, students and others not actively engaged in the workforce.

All cases were classified by clinical tumor stage (stage I: microinvasive and localized, stage II: invasion of surrounding organs or tissue, stage III: spread to distant nodes or tissue within the pelvis, stage IV: distant metastases) according to FIGO guidelines.

The national guidelines for organized cervical cancer screening recommend screening every 3 years for all women between the ages of 25 and 65 years. Age groups for *in situ* tumors were classified according to organized screening age groups. Cancer topography, morphology and behaviour were coded in accordance with the International Classification of Diseases for Oncology (ICD-O3) for the clinical data [10]. Cancer cases were recorded in strict conformity with the international standards laid down by the International Agency for Research on Cancer (IARC), the French FRANCIM network and the European Network of Cancer Registries (ENCR). Active and passive follow-up methods were used to obtain the vital status of patients. Mortality data were obtained from the Centre for epidemiology of causes of death, run by the French National Institute of Health and Medical Research (INSERM). Patient follow-up was obtained from the civil deaths register for the survival analysis. Martinique Cancer Registry studies have been the subject of a declaration to the National Authority for the protection of privacy and personal data (Commission Nationale de l’Informatique et des Libertés, CNIL).

### Statistical analysis

Patient characteristics are described as median [range] or mean with standard deviation [2] for quantitative variables, and as number (percentage) for qualitative variables. Age-standardized incidence rates were calculated using standardization on the world population. Comparisons were performed using the chi-square test or Student *t*-test, as appropriate, for quantitative variables or using Pearson’s chi-square test or Fisher’s exact test for qualitative variables, as appropriate.

Geographical distribution is described by areas and by ‘aggregated units for statistical information’ (in French, Ilot Regroupé pour l’Information Statistique, or IRIS). IRIS are geographical units of equal size used for geographical analysis. In this analysis, the type of IRIS used was the residential IRIS, based on residential areas (North and Center – South), and defined as having a population between 1800 and 5000 inhabitants. Martinique was divided into 141 IRIS [11].

For our mapping analysis, we selected the model with the lowest Deviance Information Criterion (DIC = 750.6). Bayesian analysis was used to take into account spatial autocorrelation (spatial component) and spatially unstructured overdispersion (heterogeneity component). The Bayesian models made it possible to smooth the SIRs to take into account the small sample sizes. Based on the results of the model process, SIRs were calculated for each IRIS using a Gamma Poisson model.

Overall survival was defined as the time from diagnosis to death or to the date of last follow-up. Survival rates were calculated using the Kaplan–Meier method. The cut-off date for the survival analysis was 31 December 2014. Patients who were alive after the cut-off date were censored. The median follow-up was calculated using the reverse Kaplan–Meier method. Log-rank tests were used to calculate differences according to prognostic factors. Hazard ratios (HRs) and 95% confidence intervals (CIs) were estimated to select risk factors by the Cox model. Multivariate analysis of prognostic factors was performed using Cox’s proportional hazards regression model, including all variables with a *p*-value < 0.20 by univariate analysis. For all analyses, the level of statistical significance was set at *p* < 0.05. All analyses were performed using SAS software version 9.2 (SAS Institute Inc., Cary, NC, USA) and Winbugs® 1.4 [12,13].

## Results

**Figure 1.**
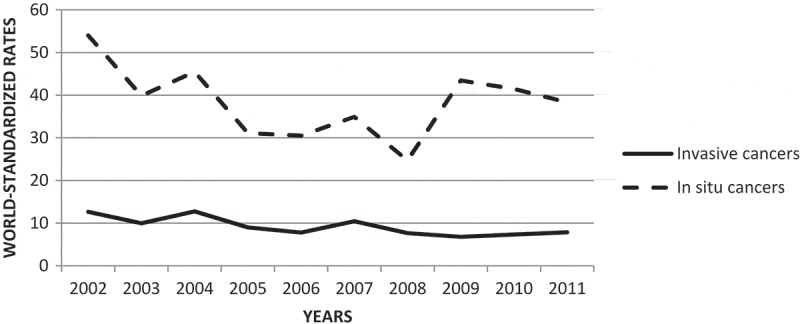
Evolution of world-standardized incidence rates for *in situ* cervical tumor and invasive cervical cancer – Martinique, French West-Indies, 2002–2011.

We examined a total of 1253 cervical cancers (947 *in situ* tumors and 306 invasive cancers) for the study period 2002–2011. Standardized incidence of cervical cancer decreased from 12.6/100,000 person-years in 2002 to 7.8/100,000 person-years in 2011. The trends in world-standardized incidence rates for *in situ* cervical tumors and invasive cervical cancer between 2002 and 2011 are presented in Figure 1.

For *in situ* tumors, median age at diagnosis was 40 years with a minimum of 16 years and a maximum of 91 years. For invasive cancers, median age at diagnosis was 58.5 years, with a minimum of 23 years and a maximum of 97 years. Socio-demographic and clinical characteristics are reported in Table 1.

**Table 1.** Sociodemographic and clinical characteristics of *in situ* and invasive cervical cancers – Martinique, French West-Indies, 2002–2011.

Characteristic	*n*	%
*In situ* tumors	947	100
Age at diagnosis		
< 25 years	36	3.8
25–44 years	611	64.5
45–64 years	243	25.7
> 65 years	57	6.0
Area		
North and Center	561	59.9
South	376	40.1
Missing	10	
Histological type		
Intraepithelial neoplasia	919	97.1
*In situ* squamous cell carcinoma	20	2.1
*In situ* adenocarcinoma	8	0.8
Invasive cancers	306	100
Age at diagnosis		
≤ 59 years	160	52.3
> 60 years	146	47.7
Area		
North and Center	187	61.5
South	117	38.5
Missing	2	
Histological type		
Squamous cell carcinoma	250	81.7
Adenocarcinoma	33	10.8
Other types	23	7.5
Invasion		
Localized tumor	201	65.7
Lymph node involvement or metastasis	74	24.2
Missing	31	10.1
FIGO stage		
I–II	186	66.7
III–IV	93	33.3
Missing	27	
Tumor size		
≤ 4 cm	93	33.2
> 4 cm	187	66.8
Unknown	26	
Professional activity		
Active	156	67.5
Inactive	75	32.5
Missing	75	

**Figure 2.**
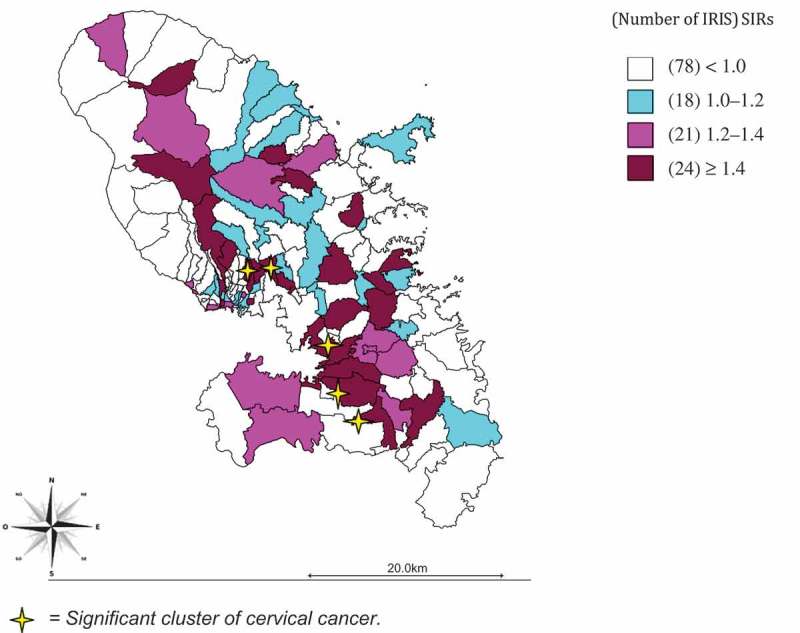
Spatial distribution by IRIS of *in situ* and invasive cervical cancer – Martinique, French West-Indies, 2002–2011. Note: There are five significant clusters of cervical cancer.

For *in situ* tumors, 90.2% of cases were within the age groups of screening programs; 36 cases (3.1%) were under 25 years old. For invasive cancers, only 2 cases were under 25 years old. Regarding invasive cancers, 81.7% of cases were squamous epithelial tumors and 65.7% were localized tumors. Among cases with data on FIGO stage (279 cases), 33.3% were FIGO stage III and IV. Among cases with data on tumor size (280 cases), tumor size up to 4 cm represented 66.8% of cases. A total of 1230 cases with geolocalization were used to map the distribution of the incidence of *in situ* and invasive cervical cancers. Five IRIS were significantly over-incident: three IRIS in the south and two IRIS in the center zone with SIRs between 1.99 [1.09; 2.89] and 2.58 [1.54; 3.63] (Figure 2.).

**Figure 3.**
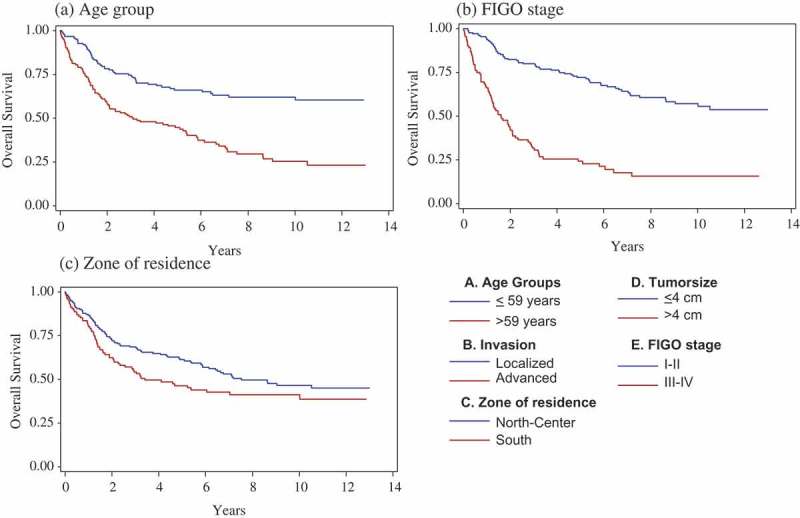
Overall crude survival of invasive cervical cancer according to (a) age group, (b) FIGO stage and (c) zone of residence – Martinique, French West-Indies, 2002–2011.

Table 2 shows the survival rates of the 306 invasive cancers. The median duration of follow-up was 8.3 years [95% CI: 7.6–9.6]. At the cut-off date, 150 women had died (49.1%) and 31 women (20.3%) were lost to follow-up. Three women had died after the cut-off date. Median survival was 6.5 years [4.9–10.0]. The overall 5-year survival rate was 55%. Figure 3 presents the overall survival curves of invasive cervical cancer (Kaplan–Meier curves). Table 3 shows the results of Cox regression analysis. By univariate analysis, age, zone of residence, tumor size, FIGO stage and invasion were statistically significant. For the multivariate analysis, we included the variables age, FIGO stage and zone of residence in the model, in view of the *p*-value by univariate analysis, and the existence of collinearity between invasion, tumor size and FIGO stage. Multivariate analysis confirmed age at diagnosis (HR = 2.15 [1.50–3.09]; *p* < 0.0001), FIGO stage (HR = 3.53 [2.50–4.99]; *p* < 0.0001) and zone of residence (HR = 1.51 [1.06–2.13]; *p* = 0.02) as risk factors.

**Table 2.** Survival rates of invasive cervical cancers – Martinique, French West-Indies, 2002–2011.

	1-year survival		3-year survival		5-year survival		10-year survival	
	%	95% CI	%	95% CI	%	95% CI	%	95% CI
Whole population	84.1	[80–88]	62.6	[57–68]	55.1	[49–61]	43.3	[37–50]
Age at diagnosis								
≤ 59 years	91	[86–95]	74	[67–81]	65	[56–74]	60	[51–69]
> 60 years	76	[69–83]	50	[42–58]	44	[36–52]	23	[14–32]
Professional activity								
Active	85	[79–91]	68	[61–75]	62	[54–70]	44	[35–53]
Inactive	87	[79–95]	61	[49–73]	46	[33–58]	36	[19–53]
Zone of residence								
North and Center	86	[81–91]	68	[61–75]	61	[54–68]	45	[36–53]
South	80	[73–87]	53	[44–62]	45	[36–54]	39	[29–49]
Histological type								
Squamous	85	[80–89]	64	[58–70]	56	[49–62]	43	[36–50]
Other types	79	[67–89]	54	[40–68]	49	[35–63]	–	–
FIGO stage								
I–II	94	[90–97]	79	[73–85]	71	[64–78]	56	[47–65]
III–IV	65	[55–75]	31	[21–41]	23	[14–32]	–	–
Tumor size								
≤ 4 cm	99	[97–100]	88	[81–95]	85	[77–93]	72	[60–84]
> 4 cm	77	[71–83]	49	[42–56]	39	[32–46]	25	[17–33]
Invasion								
Localized	90	[86–94]	73	[67–79]	67	[60–74]	53	[44–61]
Advanced	68	[57–79]	32	[21–43]	20	[10–29]	8	[0–17]

**Table 3.** Univariate and multivariate analysis using the Cox model – cervical cancer, Martinique, French West-Indies, 2002–2011.

	Univariate			Multivariate			
	HR	95% CI	*p*		HR	95% CI	*p*
Age	1.03	[1.02–1.04]	< .0001				
Age at diagnosis				Age at diagnosis			
≤ 59 years	Ref			≤ 59 years	1		
> 60 years	2.50	[1.78–3.49]	< .0001	> 60 years	2.15	[1.50–3.09]	< .0001
Professional activity							
Active	Ref						
Inactive	1.18	[0.79–1.74]	0.42				
Zone of residence				Zone of residence			
North and Center	Ref			North and Center	1		
South	1.38	[1.00–1.91]	0.049	South	1.51	[1.06–2.13]	0.02
Histological type							
Squamous	Ref						
Other types	1.13	[0.74–1.71]	0.57				
FIGO stage				FIGO stage			
I–II	Ref			I–II	1		
III–IV	4.04	[2.88–5.68]	< .0001	III–IV	3.53	[2.50–4.99]	< 0.0001
Tumor size							
≤ 4 cm	Ref						
> 4 cm	5.30	[3.22–8.71]	< .0001				
Invasion							
Localized	Ref						
Advanced	3.81	[2.71–5.35]	< .0001				

## Discussion

Here, we report the results of an observational study conducted on data from a population-based cancer registry in the French West-Indies. Across the Caribbean, few epidemiologic studies are available on cervical cancer incidence and survival [14]. Over the last decade, the burden of cervical cancer has been established and several National Cancer Plans have been presented. Population-based cancer registries are important in the public health surveillance system to better understand trends in cancer incidence and prognostic factors for cancer survival [15].

Latin America and the Caribbean have one of the highest incidence rates worldwide for cervical cancer [15]. To improve our knowledge of risk factors for cancer, the National Cancer Plan 2014–2019 provides new opportunities to launch prognostic and epidemiological studies on cervical cancer through specific research programs in the French West-Indies.

In order to contribute to the international epidemiology on cervical cancer, we conducted this study using data from a population-based cancer registry, to ensure the quality and exhaustiveness of data for our cases. In our study, we included a total of 1253 cervical cancers, comprising 947 *in situ* tumors and 306 invasive cancers, over the period 2002–2011. *In situ* tumors were three times more incident than invasive cancers. From 2002 to 2011, a decrease in the incidence of cervical cancer was observed, with the standardized incidence decreasing from 12.6/100,000 to 7.8/100,000 person-years for invasive cancers. Despite a higher incidence in Martinique, these trends are in line with French national data and are linked to the improvement in hygiene conditions and the development of individual and organized cervical screening by Pap smear testing [16]. Further socio-environmental studies are warranted to explore these preliminary results. The inclusion of vaccination against the papillomavirus in vaccination schedules supported by the National Health Insurance since 2007 may help to decrease the incidence of cervical cancer in the future. Action 1.2 of the National Cancer Plan 2014–2019 targets an improvement in the coverage rate of this vaccination (60% of coverage expected) by strengthening the mobilization of general practitioners and health authorities. The National Cancer Plan therefore aims to increase access to vaccination against HPV, which is expected to have a longer-term impact, with free access for the girls concerned [4].

In our study, 90% of *in situ* tumors were diagnosed in women aged 25–65 years, corresponding to the target age of organized screening programs. However, 38 women were aged less than 25 years and were not eligible to benefit from organized screening. Fortunately, only two of these were diagnosed with invasive cancer. Organized and individual screening coverage reached 41.0% in Martinique. A study evaluating the organized screening program for cervical cancer in Martinique reported a low participation rate (11.9%). Although this coverage is still insufficient, the positive impact of this program has been demonstrated [2,17]. The involvement of general practitioners, midwives and gynecologists is essential to detect and inform women in the target age group.

In our sample, the majority of invasive cancers (66.7%) were diagnosed at an early stage (FIGO stage I–II), and this is likely due to the introduction in 1991 of a pilot screening program on the island. Nevertheless, a large proportion of tumors (66.8%) were found to have a tumor size up to 4 cm. For the overall population, the 5-year survival and the 10-year survival rates were respectively 55% and 43%. These rates are well below the national rates of 63% and 54% [18], and this can likely be explained by ethnic, socio-economic and etiologic differences in cancer survival. In the US, significant survival differences have been observed, with a higher incidence in African American women [19,20]. Lin et al. also showed that the risk of death for cervical cancer increased with decreasing socio-economic status [20]. The main etiological factor for the development for this type of cancer is HPV; among the many known genotypes, HPV 16 and 18 are identified in 70% of invasive cervical cancers in Western countries [9,16]. However, for the Caribbean area, genotyping is in process and could be different [21–23].

The survival rate is one of the key indicators of the effectiveness of the system of cancer care. However, implementation of organized screening can have paradoxical consequences on survival. According to the literature, cancers diagnosed at the invasive stage are less numerous but include a higher proportion of cancers with poor prognosis, inducing a decrease in survival. Thus, invasive cancer of the cervix saw its survival decrease from 68% to 64% between 1990 and 2002, with the development of individual screening in France [18]. In our study, univariate analysis indicated better survival when cervical cancer was detected at an early stage. Survival was strongly associated with the tumor size, the FIGO stage and the state of invasion (*p* < 0.0001). These results are in line with the objectives of organized screening. Age at diagnosis is obviously a significant prognostic factor for survival (*p* < 0.0001). The zone of residence was also found to be a prognostic factor for survival (*p* = 0.049) and needs to be explored in geographical analysis while also taking into account socio-economic determinants. A specific index of deprivation for the French West-Indies could help to perform such analyses in the future.

Multivariate analysis confirmed age at diagnosis (*p* < 0.0001), FIGO stage (*p* < 0.0001) and zone of residence (*p* = 0.02) as prognostic factors (Table 3). In a recent study from the Burgundy Cancer Registry, significant prognostic factors identified were age, advanced stage and non-accurate follow-up (screening) [24]. The effect of the zone of residence on survival could be linked to access to cancer screening and cancer care in Martinique and will be explored in further studies.

### Strengths and weaknesses of the study

The strengths of this study include the high quality of the data. The use of data from this population-based cancer registry ensures exhaustiveness of the cases included in our study, therefore ensuring good representativeness of cervical cancer in Martinique. Rigorous data quality control in compliance with the recommendations of the IARC (i.e. regular checking to ensure the complete follow-up for each woman, cross-referencing of information sources) ensures that the level of missing clinical and pathological data is kept to a minimum. This study also used population census data from the French National Institute for Statistics and Economic Research to ensure the robustness of the incidence results.

The methods used in this paper could easily be applied in other regions with similar infrastructures (i.e. an existing cancer registry, and a national body that carries out population censuses on a nationwide scale). Therefore, we believe that our study can be generalized by respecting these minimum conditions. Nonetheless, while these results are representative of the entire population of Martinique, local specificities (in terms of genetic or environmental factors, for example) may preclude extrapolation at the international level.

Conversely, this study suffers from two main limitations. Firstly, we lacked sufficient data to evaluate potential social inequalities in healthcare and health status. Indeed, income levels and other socio-economic indicators were not collected in the Martinique Cancer Registry, and the high rate of missing data for the only available socio-economic indicator (namely, professional activity) may have affected the validity of these results. Improved data collection for variables relating to socio-economic status could help to better identify high-risk groups in our region. Secondly, unfortunately, data relating to sexual activity (including mean age at first sexual intercourse, use of contraception, number of sexual partners and co-infection with sexual pathogens) were not collected. These risk factors may contribute to explaining the high incidence rate observed in the Caribbean zone and should be taken into account in future research.

## Conclusion

Our results are representative of the population of Martinique and will help develop cervical cancer prevention strategies in Martinique. Data from population-based cancer registries represent a gold standard and are widely used in the evaluation and monitoring of screening programs. The strategic Cancer Plan in the French West-Indies will focus on infectious cancers to explore in greater depth the risk factors for cervical cancer, and the role of the HPV genotype in cohort studies. Regarding the epidemiological data, further studies will be conducted to describe the profile of cervical cancer in young women in Martinique and in the French West-Indies. Geographical and socio-economic inequalities need to be explored to better characterize the prognostic factors for cervical cancer. Clearly, cervical cancer needs to be diagnosed at an early stage in order to improve survival. Our results could allow cervical cancer screening programs to clearly identify geographical areas that would benefit from targeted interventions with a view to reducing incidence and mortality of cervical cancer in the French West-Indies and the Caribbean. These data could help to perform assessment of screening programs in the Caribbean.

## Paper context

Cancer is a leading cause of premature death, especially in women. The Caribbean ranks seventh among world regions most affected by cervical cancer. By describing the epidemiology of cervical cancer, we explore disparities in incidence and mortality based on data from a population-based cancer registry in Martinique. Our results could allow cervical cancer screening programs to identify specific factors amenable to targeted intervention, and define an action framework to reduce the burden of cervical cancer in Martinique.
